# Engineering Nanomedicine for Non-Viral RNA-Based Gene Therapy of Glioblastoma

**DOI:** 10.3390/pharmaceutics16040482

**Published:** 2024-04-01

**Authors:** Wenya He, Ningyang Wang, Yaping Wang, Mengyao Liu, Qian Qing, Qihang Su, Yan Zou, Yang Liu

**Affiliations:** 1 School of Pharmacy, Henan University, Kaifeng 475004, China; wyhe@henu.edu.cn (W.H.);; 2 Translational Medicine Center, Huaihe Hospital of Henan University, Henan University, Kaifeng 475004, China; 3 Henan Key Laboratory of Brain Targeted Bio-Nanomedicine, School of Life Sciences & School of Pharmacy, Henan University, Kaifeng 475004, China; 4 Henan International Joint Laboratory of Nanobiomedicine, School of Life Sciences, Henan University, Kaifeng 475004, China

**Keywords:** glioblastoma, nanomedicine, gene therapy, RNA therapy, non-viral nano-delivery

## Abstract

Glioblastoma multiforme (GBM) is the most common type of malignant tumor of the central nervous system, characterized by aggressiveness, genetic instability, heterogenesis, and unpredictable clinical behavior. Disappointing results from the current clinical therapeutic methods have fueled a search for new therapeutic targets and treatment modalities. GBM is characterized by various genetic alterations, and RNA-based gene therapy has raised particular attention in GBM therapy. Here, we review the recent advances in engineered non-viral nanocarriers for RNA drug delivery to treat GBM. Therapeutic strategies concerning the brain-targeted delivery of various RNA drugs involving siRNA, microRNA, mRNA, ASO, and short-length RNA and the therapeutical mechanisms of these drugs to tackle the challenges of chemo-/radiotherapy resistance, recurrence, and incurable stem cell-like tumor cells of GBM are herein outlined. We also highlight the progress, prospects, and remaining challenges of non-viral nanocarriers-mediated RNA-based gene therapy.

## 1. Introduction

Glioblastoma multiforme (GBM) is the most aggressive type of brain tumor without an efficient cure. Currently, the clinical standard treatment for GBM, which consists of maximal surgical resection followed by radiation and temozolomide (TMZ) chemotherapy, cannot provide satisfactory therapeutic effects. The median survival time of GBM patients is only 15–16 months, and the 5-year overall survival rate is less than 5% [1,2,3]. The high mortality of GBM is due to the consequence of many contributing factors. On the one hand, owing to the highly diffusive growth and aggressive nature of GBM, surgical removal cannot completely resect the tumor, which leads to a high tendency of recurrence. On the other hand, GBM is often resistant to radiation and/or chemotherapy, and the existence of the blood–brain barrier (BBB) further limits the therapeutic effect of chemotherapy. Moreover, radiotherapy and chemotherapy often induce severe side effects and, as a result of their low selectivity to tumor cells, kill normal cells as well [4,5,6]. These problems have compelled scientists to develop alternative therapeutic approaches for GBM.

With an improved understanding of the molecular biology of GBM, the treatment failure of current strategies is found to be closely correlated with the unique molecular characteristics of GBM, e.g., the presence of stem cell-like tumor cells, intratumoral heterogeneity, tumor immune escape, and immune suppressive status of the tumor microenvironment (TME). These encouraging findings have extended the therapeutic strategies to target not only tumor cells but also the TME, tumor-intrinsic dominant signaling pathways, and tumor immune modulation [7,8,9,10]. Various molecularly targeted approaches such as immunotherapy and gene therapy have been actively investigated and tested in clinical trials for GBM [11,12]. Gene therapy is now bringing a revolution to cancer treatment. In general, the definition of cancer gene therapy is the delivery of specific genetic material (DNAs or RNAs) to modify the gene at the epigenetic, genetic, and transcriptional levels and control tumor growth or lead to the locoregional production of a product that could prove detrimental to the tumor cells [13]. Actually, GBM gene therapy can be extended to immunotherapy along with the emergence of RNA vaccines as well as GBM therapy via modulation of a tumor-immunosuppressive microenvironment. GBM is characterized by various genetic risks, such as mutations in TP53, PTEN, EGFR, and their downstream signaling pathways, and these potential molecular therapeutic targets have aroused extensive interest in research [14].

RNA-based gene therapy for GBM has received particular attention since it functions as a bridge between DNAs and proteins. Theoretically, RNA molecules such as ASOs, siRNAs, and microRNAs can not only directly target any interest gene through Watson–Crick base pairing with the complementary nucleotide sequence but also can block the expression of the specific proteins at the transcriptional level. Moreover, gene therapy mediated by RNA may not cause irreversible genome changes and induce genetic risks like DNA-based therapeutics [15,16]. One of the important factors for RNA-based therapy is the intracellular delivery of RNA molecules. RNAs are negatively charged, have a large molecule size, and are susceptible to RNases present in both the blood circulation and tissues, which makes it difficult for RNA to enter cells efficiently and function on its own [17]. Viral vectors are efficient carriers that can protect RNA from degradation, maximize delivery to on-target cells, and generate successful clinical outcomes in some diseases. Viral-vector-mediated RNA transfection strategies have been tested in clinical trials for GBM therapy (NCT00002824, NCT00031083, and NCT00870181). However, the effectiveness of viral vectors is hampered by the risk of immunotoxicity, insertional mutagenesis, payload size constraints, complications involved in upscaling, and expensive vector production [18,19]. Concurrent advances in the development of synthetic materials that encapsulate RNA molecules, such as polymers, liposomes, and lipid nanoparticles (LNP), have invigorated research into the engineering of non-viral gene delivery systems [20]. Synthetic non-viral nanoparticulate gene delivery systems have been extensively investigated for GBM therapy during the past decades. Herein, we review the recent advances in RNA-based non-viral nanoparticulate therapeutics for GBM therapy. We outline the brain-targeted delivery of RNA drugs involving siRNA, microRNA, mRNA, ASO, and shRNA by using non-viral nanoparticulate carriers such as polymeric nanoparticles, liposomes, extracellular vesicles (e.g., exosomes), as well as inorganic nanoparticles for GBM therapy. We focus on the targeting strategies of the designed RNA-based nanomedicines for BBB penetration and GBM targeting and the therapeutical mechanism of these RNA drugs for settling the challenges of chemo-/radiotherapy resistance, recurrence, and incurable stem cell-like tumor cells that confront current GBM therapy. The clinical translation of the non-viral nanoparticulate RNA-based GBM therapies and their remaining challenges are also highlighted.

## 2. Recent Advances in the Development of Non-Viral Nanomedicine for RNA-Based GBM Gene Therapy

Non-viral nanoparticles (NPs) have garnered considerable interest in brain drug delivery thanks to their distinctive structures and multifunctional capabilities. For successful crossing of the blood–brain barrier (BBB), the physicochemical characteristics of the nanomaterials, such as size, shape, and surface charge, must be carefully tailored. Particle size, in particular, should be given primary consideration. NPs of varying sizes exhibit distinct pharmacokinetics in vivo and tend to accumulate in different organs at different rates. In general, NPs smaller than 5 nm in diameter are considered able to cross the BBB through passive diffusion. However, their short blood circulation time and rapid renal clearance lower their residence time in brain microvessels and therefore their brain delivery. NPs larger than 100 nm undergo rapid clearance by macrophage phagocytosis in the liver and spleen, which also shortens their blood circulation, hinders their penetration across the BBB, and induces organ toxicity concerns [21]. Shape is another key factor affecting the pharmacokinetics and BBB permeability of the NPs owing to their differences in contact quality and contact areas with the target cells in the BBB. It has been reported that the rod-shaped NPs display stronger binding ability toward the BBB endothelium cells than the spherical NPs [22]. The proper length/width aspect ratio is another factor that determines the brain uptake efficiency of NPs. Zeta potential can also have an impact on the BBB-crossing ability of NPs. The positively charged NPs can more easily cross the BBB through the adsorptive-mediated transcytosis relative to the negative or neutral NPs, while they display higher cytotoxicity and tendency to absorb the serum protein in vivo, which results in a faster plasma clearance rate [23]. Based on the above discussions, it is essential to consider the benefit-to-risk ratio and adjust the physiological properties of NPs so they may easily penetrate the BBB with low side effects. It has been demonstrated that proper surface functionalization (e.g., actively targeting ligand Angiopep-2, ApoE, and RVG peptide) is the most important determinant for BBB crossing [24,25], and particle size seems to have little impact on the BBB penetration of the functionalized NPs. Additionally, surface coating directly correlates with their surface charge and pharmacokinetics.

Apart from the physiological properties, the administration route of the NPs should be considered for brain-targeted drug delivery. Drug delivery to the central nervous system (CNS) can be implemented by several administration routes, including local delivery, intranasal route, and systemic delivery. Local delivery is directly delivering the NPs to the brain by injection with a catheter, which requires surgery and is therefore highly invasive. Intranasal delivery allows direct entry of the NPs to the CNS mainly via the olfactory pathway and the trigeminal pathway. Recently, the intranasal route has been recognized as an attractive way for brain delivery of drugs, but its efficacy largely depends on the state of the nasal mucosa, which has shown differences between mice and humans [26,27]. Therefore, despite the difficulty of crossing the BBB, the most popular and well-studied delivery route for nanoparticulate RNA drugs remains the systemic pathway. Below, we discuss the well-designed RNA delivery nanoplatforms for GBM therapy.

### 2.1. siRNA-Based Nano-Therapeutics for GBM

siRNA, also known as small interfering RNA, is an exogenous double-stranded RNA molecule consisting of 22–25 nucleotide pairs. Upon binding to homologous mRNA, siRNA can mediate the silencing of relevant genes, a process known as RNA interference (RNAi) [28]. Starting from the appearance of the first siRNA drug Patisiran in 2018, six siRNA drugs have been approved for market use [29,30]. NU-0129, a spherical nucleic acid that consists of gold nanoparticle cores covalently conjugated with radially oriented and densely packed siRNA oligonucleotides for Bcl2L12, has entered early phase I clinical trials for GBM therapy (NCT03020017), which is encouraging for brain tumor siRNA therapy. In this section, we review the representative works in nanomedicine-mediated siRNA delivery for GBM therapy.

**Figure 1:**
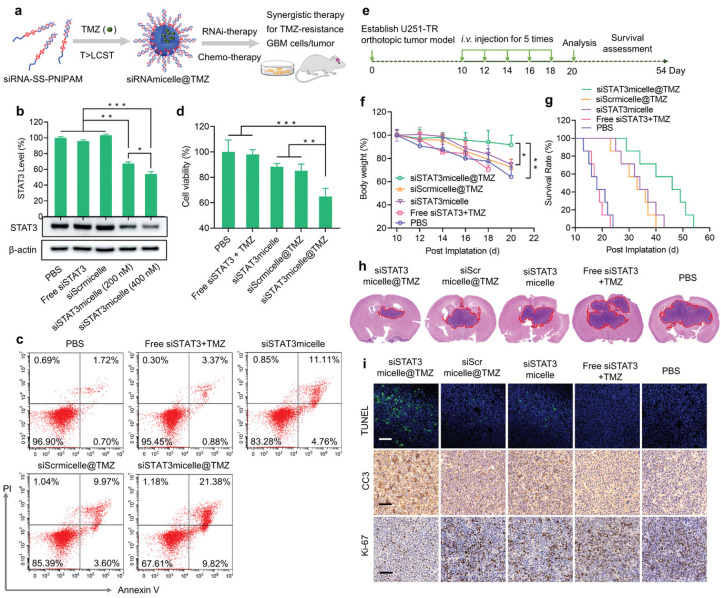
Demonstration that the TMZ-loaded nanomicelles achieved efficient therapeutic effects in the TMZ-resistant GBM animal models. (**a**) Illustration of the formation of siRNAmicelle@TMZ and its function. (**b**) STAT3 gene silencing evaluation of the nanomicelles by RT-PCR and Western blot analysis. (**c**) Apoptosis of TMZ-resistant U251-TR cells after incubation with siSTAT3micelle@TMZ. (**d**) Cell cytotoxicity evaluation of the nanomicelle toward U251-TR cells. (**e**) Schematic of the experimental timeline. (**f**) Body weight changes and (**g**) survival rates of the tumor-bearing mice after nanomicelles treatment. (**h**) H&E images of U251-TR GBM brain excised from mice on day 20 posttreatment. (**i**) Immunohistochemistry in tumor tissues from mice treated with siSTAT3micelle@TMZ or controls. *n* = 3, * *p* < 0.05, ** *p* < 0.01, *** *p* < 0.001. (Scale bars: 200 μm for TUNEL images and 100 μm for CC3, Ki-67 images.) (Reprinted with permission from ref. [32], Copyright 2021, Wiley).

The polo-like kinase (PLK1) is recognized as a potent oncogene that plays a key role in the survival and proliferation of GBM [31]. Nanomicelles for targeted delivery of PLK1-siRNA for GBM therapy were proposed by Zheng et al. The nanomicelles were prepared by the self-assembly of siRNA-disulfide-poly(N-isopropylacrylamide) diblock copolymers [32]. The obtained micelles can protect the siRNA from nuclease degradation in blood and possess prolonged blood circulation with an elimination half-life (t1/2, β) of ≈31 min, which is markedly longer than that of free siRNAs (t1/2, β = 4 min) when administered intravenously. Efficient BBB penetration and GBM accumulation were obtained via the cell-membrane-bound scavenger receptor-mediated mechanisms. A potent anti-GBM effect of the nanomicelles was achieved in the U87 MG orthotopic GBM tumor mouse models without inducing noticeable charge-associated adverse effects. Moreover, the inner core of the nanomicelles could efficiently encapsulate chemical drugs and TMZ and realize drug release in the presence of the intracellular glutathione GSH. By codelivery with the siRNA target at the oncogenic and tolerogenic signal transducer and activator of transcription 3 (STAT3), the TMZ-loaded nano-micelles (siRNAmicelle@TMZ) achieved efficient therapeutic effects in TMZ-resistant GBM animal models (Figure 1). Additionally, due to being non-cationic, the siRNA-loaded nanomicelles exert no charge-associated toxicity. This is the first report on siRNA-decorated nanomicelles (polymeric spherical nucleic acid) for GBM therapy. Similarly, to avoid the systemic toxicity of the cationic components of the transfection agents, Gregory et al. [33] engineered a cation-free synthetic protein nanoparticle based on polymerized human serum albumin equipped with the tissue-penetrating peptide iRGD for brain-targeted delivery of STAT3-siRNA. Benefitting from the specific recognition of the albumin and SPARC and gp60 receptors on the BBB endothelium and GBM cell surface as well as the recognition of iRGD with the overexpressed αvβ3 and αvβ5 integrins on the GBM cells, the obtained nanomedicine could cross the BBB and be distributed throughout the tumor volume without the use of invasive surgical procedures via intravenous (i.v.) injection. Efficient downregulation of STAT3 was achieved both in vitro and in vivo. Apart from tumor progression, the STAT3 pathway is also involved in the tumor evasion of the immune system. When combined with the current standard focused radiotherapy, the obtained nanomedicine not only displayed combinatory GBM regression but also primed the immune system to develop anti-GBM immunological memory, which prevented the GBM tumor rechallenge.

Increased expression and activity of the DNA repair protein O6-methylguanine-DNA methyltransferase (MGMT) is reported to be one of the most important mechanisms accounting for TMZ resistance of GBM tumor cells [34,35]. Wang et al. [36] reported the development of an iron oxide nanoparticle system functionalized with a GBM-targeting ligand chlorotoxin for targeted delivery of MGMT-siRNAs for GBM therapy. The obtained nanoparticles could cross the BBB, target tumors via tail vein injection, and sensitize both GBM cells and GBM stem-like cells to TMZ by suppressing the expression of MGMT. Effective tumor inhibition and prolonged survival were found in the orthotopic serially passaged patient-derived GBM xenografts mouse model. Moreover, the intrinsic superparamagnetism of the iron oxide nanocore enabled nanoparticle MRI imaging capacity for non-invasive tumor imaging and treatment response monitoring.

Metastasis-associated lung adenocarcinoma transcript 1 (MALAT1) is one of the cancer-promoting long non-coding RNAs that are overexpressed in human glioma tissue and positively correlated with tumor malignancy and poor patient survival [37]. Kim et al. [38] constructed a nanocomplex based on the cationic liposome decorated with a monoclonal antibody (TfRscFv) to deliver anti-MALAT1 siRNA for RNAi therapy of GBM. The TfRscFv mediates both the active crossing of the BBB and tumor targeting of the nanocomplex following i.v. systemic injection. Experimental results showed that silencing of MALAT1 could not only inhibit the proliferation and migration of GBM cells but also increase the sensitivity of GBM cells to chemical drugs including TMZ. In the orthotopic GBM mouse model, tumor inhibition and prolonged life survival were achieved with concurrent treatment of TMZ and nanocomplex-mediated silencing of MALAT1.

Apart from chemo-resistance, radiotherapy resistance is another significant clinical challenge for GBM therapy. Upregulation of Cofilin-1 (CFL1), a tumor progression marker deemed a “metastasis switch”, is one of the mechanisms by which tumor cells develop radiation resistance [39]. Tang et al. [40] developed X-ray-triggered nanocapsules based on selenium-engineered mesoporous silica for delivery of CFL1-siRNA (siCFL1) to treat radiotherapy-resistant GBM. siCFL1 was incorporated into the amino diselenide-bridged silica framework via electrostatic interaction and then coated with a micelle-like polymeric shell decorated with targeting ligand Angiopep-2. The obtained nanomedicine possessed a blood elimination half-life (t1/2) of approximately 4.2 h following i.v. injection, which was longer than that of free siCFL1 and favored their GBM tumor location. Upon exposure to X-ray irradiation, a burst of intracellular ROS cleaved the diselenide-bridged silica backbones of the nanomedicine, resulting in silica matrix collapse and rapid siCFL1 release, leading to transcription interference and knockdown of CFL1 protein expression. Together with the transformation of metronidazole (grafted in the polymeric shell of the nanocapsules) into aminoimidazole in the hypoxic condition that resulted from the production of ROS, which would enhance the radiotherapy-triggered tumor cytolysis by fixing ROS-induced DNA destruction to inhibit DNA repair, an efficient therapeutic anti-radiotherapy-resistant GBM effect was achieved both in vitro and in vivo.

Ferroptosis is a newly discovered form of regulated cell death that is induced by excessive lipid peroxidation. In essence, ferroptosis is an iron-dependent cell death and involves the decrease in antioxidant capacity and accumulation of lipid reactive oxygen species (ROS) in cells [41,42]. Glutathione peroxidase 4 (GPX4) is a key regulator of ferroptosis, and agents that inhibit the activity of GPX4 have been developed for cancer therapy. Recent studies have reported that dihydroorotate dehydrogenase (DHODH) would compensate for the inhibition of GPX4 to avoid cell ferroptosis and thus could serve as a new therapeutic target for ferroptosis [43]. Based on these discussions, Li’s group [44] designed a composite therapeutic platform (MNP@BQR@ANG-EXO-siGPX4) based on the exosome-conjugated magnetic nanoparticles for brain-targeted co-delivery of GPX4-siRNA (siGPX4) and DHODH inhibitor (BQR) for GBM therapy. The exosome was derived from the human mesenchymal stem cells (hMSCs) and functionalized with Angiopep-2 (ANG) peptide as an ANG and Lamp2b fusion protein on the exosome surface for GBM targeting. SiGPX4 was encapsulated into the exosome via electroporation. A Fe_3_O_4_ core covering a mesoporous silica shell conjugated with a CD63 antibody and loaded with BQR was conjugated onto the exosome through the binding of the CD63 antibody with the CD63 antigens expressed on the exosome surface. The Fe_3_O_4_ core in the obtained platform not only could guide the conjugate to reach the brain with the assistance of the local magnet installed in the mouse head following i.v. injection but also release Fe^2+^ in the acidic condition in the tumor cells to trigger the production of ROS. With systemic administration, synergistic ferroptosis therapy of GBM is achieved by the combined triple actions of the disintegration of DHODH and the GPX4 ferroptosis defense axis with Fe_3_O_4_ nanoparticle-mediated Fe^2+^ release.

### 2.2. mRNA-Based Nano-Therapeutics for GBM

Messenger RNA (mRNA) is a transient carrier that transfers genetic information from DNA to ribosomes, where that information can be translated into proteins. By delivering mRNAs that express antigens of infectious diseases or cancers, gene-editing components, or disease-related therapeutic proteins, various clinical applications can be achieved [45,46]. Recently, the successes in the development of the two coronavirus 2019 (COVID-19) mRNA vaccines (Moderna mRNA-1273 and Pfizer/BioNTech BNT162b2) have fueled a renewed and intense research interest in mRNA engineering and delivery, offering the promise of clinical translation of various mRNA-based therapies. In GBM therapy, mRNA-based gene therapy has been extensively investigated in preclinical and clinical research. Apart from targeting oncogenes to induce GBM tumor inhibition, mRNA-based therapeutics have been used for antigens delivery to trigger anti-tumor immunity and remodeling the immunosuppressive tumor microenvironment to enhance anti-tumor immunotherapy. In this part, we will review the representative works in this area.

Tumor suppressor gene PTEN (phosphatase and tensin homolog) deletions and mutations are frequent events in GBM and are associated with therapeutic resistance [47]. Restoration of PTEN function by mRNA-based approaches are promising therapeutic opportunity for GBM treatment. Our group [48] adopted the BBB endothelium and GBM-targeting ligand (ApoE peptide)-functionalized biomimetic nanoparticle platform for selective delivery of PTEN-mRNA for orthotopic GBM therapy. The obtained nanoparticles could protect PTEN-mRNA from degradation in blood circulation following i.v. injection and could efficiently cross the BBB and target GBM via the recognition of the ApoE-peptide and the low-density lipoprotein receptor family expressed on the cell surface. Elevated expression of PTEN protein and the attenuation of the PI3K-AKT signaling pathway in the tumor tissues was found in the GBM-bearing mouse that received five doses of nanomedicine treatment, which resulted in a remarkable extension of survival.

Recently, extracellular vesicles (e.g., exosomes) have emerged as promising carriers for nucleic-acid-based therapeutics owing to their biocompatibility and long blood circulation, and some have shown an innate capability to cross the BBB [49]. Yang et al. [50] developed a cellular nanoporation (CNP) method for the large-scale production of extracellular vesicles (EVs) encapsulating PTEN-mRNAs for GBM therapy. The CNP system could not only promote the transfection of the plasmid that carries PTEN and the GBM-targeting ligand nucleotide sequences into the transfected cells but also promote enhanced EVs production and secretion via a mechanism of upregulation of heat-shock proteins (HSPs) and elevated intracellular [Ca^2+^]. The GBM-targeting peptide used for U87 MG cell targeting, CDX peptide (FKESWREARGTRIERG), and the peptide CREKA for GL261 cell targeting were functionalized onto the EVs surface via incorporation into the N terminus of CD47, an EVs transmembrane protein. In comparison to other production strategies, the CNP method produced up to 50-fold more exosomes and a more than 103-fold increase in exosomal mRNA transcripts, even from cells with low basal levels of exosome secretion. The anti-tumor effect of the PTEN-mRNA loaded exosomes was evaluated in both immunodeficient and immune-competent PTEN-deficient glioma mouse models, and robust tumor inhibition and increased survival were obtained.

**Figure 2:**
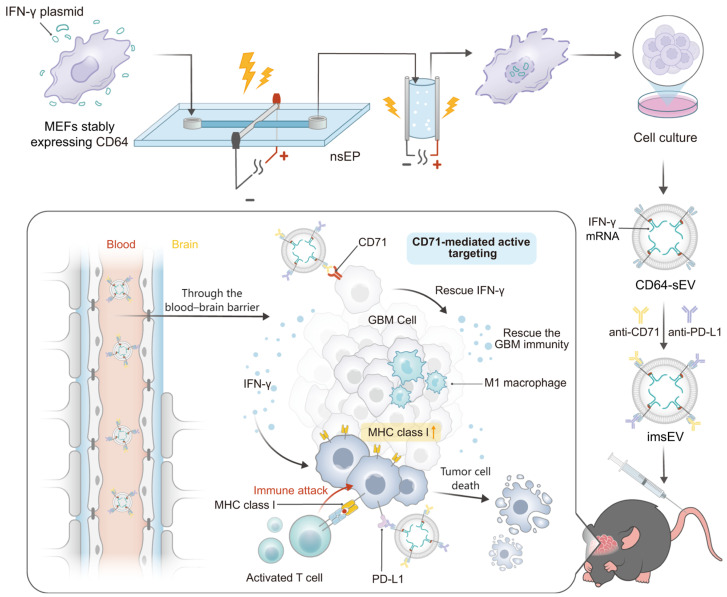
Illustration of the large-scale production and therapeutic mechanisms of imsEV. (Reprinted with permission from ref. [52], Copyright 2023, Springer Nature).

Overexpression of immune checkpoint protein PD-L1 and downregulation of major histocompatibility complex class I (MHC-I) as well as other features of the tumor microenvironment create an immunosuppressive environment for GBM that limits the effectiveness of immunotherapies [51]. Dong et al. [52] engineered IFN-γ mRNA-encapsulating small extracellular vesicles (sEVs) overexpressing CD64 on their surfaces for GBM immune therapy. As CD64 is an Fcγ receptor that can bind to the constant region of IgG heavy chain, GBM-targeting ligand anti-74 and anti-PD-L1 antibodies can be docked onto the sEVs surface. The resulting immunogenic sEVs (imsEV) preferentially targeted glioblastoma cells following i.v. injection and generated IFN-γ release in the TME. The released IFN-γ restored the MHC-I expression, modulated the function of immune cells, and, together with the anti-PD-L1 antibodies, reprogramed the immune microenvironment of the brain tumor from an immunosuppressive to an immune-stimulating phenotype. In orthotopic GBM mouse models, including the intrinsically immune-resistant mouse tumor models, a potent therapeutic effect was achieved after treatment with imsEV (Figure 2).

**Figure 3:**
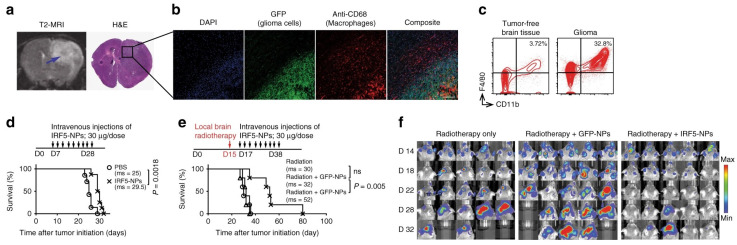
Illustration of the macrophage reprogramming by the IRF5/IKKβ-encoding nanoparticles improves the outcome of radiotherapy in glioblastoma. (**a**) T2-mode MRI scan and histological staining of the glioblastoma-bearing mouse brain. Arrow indicates the tumor region. (**b**) Confocal microscopy of CD68+ TAMs (M2 phenotype) infiltrating the glioma margin. Scale bar: 300 μm. (**c**) Flow cytometry analysis of macrophage (F4/80+, CD11b+) populations in healthy brain tissue versus glioma. Kaplan–Meier survival curves of GBM-bearing mice receiving IRF5/IKKβ treatments alone (**d**) or combined with radiotherapy (**e**). Ms, median survival. (**f**) Sequential bioluminescence imaging of tumor progression. (Reprinted with permission from ref. [54], Copyright 2019, Springer Nature).

Tumor-associated macrophages (TAMs) are a population of immune cells in GBM that comprise up to 50 % of the tumor mass. TAMs are highly plastic and can be polarized into two major subtypes, namely the antitumor M1 (TAM1) and the pro-tumor M2 (TAM2) during tumor progression. TAM2 is an important driving factor in immunosuppressive TME and strategies that can reprogram the TAM2 to TAM1 phenotype can not only lift the Immunosuppressive constraints and elicit anti-tumor immunity but also augment the chemo-/radiotherapy efficacy [53]. Zhang et al. [54] developed a TAMs-targeted nanocarrier for the delivery of in vitro-transcribed mRNA encoding M1-polarizing transcription factors to reprogram TAMs for cancer immunotherapy. The mRNA molecules encode IRF5 and IKKβ, which imprint TAMs with a potent proinflammatory and anti-tumor M1 phenotype, and were complexed with a positively charged polymer cationic poly(β-amino ester) (PbAE) and then functionalized with a macrophage-targeting ligand Di-mannose to form the nanoparticles (IRF/IKKβ NP). The designed nanoparticles could efficiently program the M2 phenotype macrophages into the M1 phenotype and reverse the immunosuppressive tumor microenvironment. In the PDG glioma mouse model, IRF/IKKβ NP treatments suppressed the tumor progression following intravenous infusion of nine doses (three doses/week for three weeks), and significant tumor inhibition was obtained when combining the nanoparticles treatment with the standard-of-care radiotherapy (Figure 3).

mRNA-based vaccines have become a promising platform for cancer immunotherapy due to their high potency, safe administration, rapid development potential, and cost-effective manufacturing. To date, several mRNA-dendritic cell vaccines have entered clinical trials (NCT00846456, NCT02808364, NCT00890032, NCT03548571, NCT00639639, NCT00626483, NCT03927222, NCT03688178, NCT02465268, NCT02366728, etc.) for GBM therapy. In a recent clinical trial launched by Zhu et al. [55], a 37-year-old GBM patient was treated with combination immunotherapy consisting of DC vaccines, anti-programmed death-1 (anti-PD-1), and poly I: C as well as the chemotherapeutic agent cyclophosphamide following the standard resection and chemo-/radiotherapy. The DC vaccines were loaded with either mRNA-tumor associated antigens (TAA), mRNA-neoantigens, or hypochlorous acid (HOCl)-oxidized tumor lysates aimed at complementing each other in promoting vaccine efficacy. The treatment was carried out from 17 October 2017 to 4 May 2022, and robust antitumor CD4^+^ and CD8^+^ T-cell responses were triggered with no immunotherapy-related adverse events observed during the treatment. The patient remains free of disease progression for 69 months. These exciting clinical results indicated that developing mRNA-based nanovaccines for GBM therapy is highly promising, and we hope the discussions here will inspire more researchers to take up the investigation of efficient nanoparticulate GBM mRNA vaccines.

### 2.3. microRNA-Based Nano-Therapeutics for GBM

microRNAs are a class of single-stranded RNA molecules with a length of about 21~23 nucleotides. microRNA can regulate gene expression post transcriptionally by mediating gene silencing or prompt target mRNA degradation. microRNAs affect numerous cancer-related signaling pathways such as differentiation, proliferation, migration, and apoptosis and are potential therapeutic targets [56]. Dysregulation of the specific microRNAs has been widely associated with GBM events, and related studies have been carried out during the past decades.

Lang et al. [57] screened a panel of eight microRNAs (miRs) against five glioma stem cells (GSCs) and identified miR-124a as an effective antiglioma miR against GSCs. They used lentivirus vectors containing miR-124a to transduce the bone-marrow-derived mesenchymal stem cells (MSCs) and isolated exosomes carrying miR-124a (Exo-miR124) from the medium. Both in vitro and in vivo experimental results showed that the obtained Exo-miR124 could efficiently inhibit the proliferation of the GBM cells and prolong the survival of the tumor-bearing mouse with systemic administration.

miR-21 is a potent oncogene that is overexpressed in GBM, which targets several genes such as PDCD4, TIMP3, and RECK and is a key regulator of cell apoptotic and metastatic pathways [58]. Monfared et al. [59] designed a sponge sequence against miR-21 (miR-21-sponge) and packaged the miR-21-sponge into exosomes of the HEK-293T cells. The obtained exosomes could suppress miR-21, upregulate the miR-21 target genes, and inhibit the proliferation of the GBM cells. In the orthotopic GBM mouse model, stereotaxically administrating the exosomes into the mouse brain led to a significant reduction in tumor growth.

**Figure 4:**
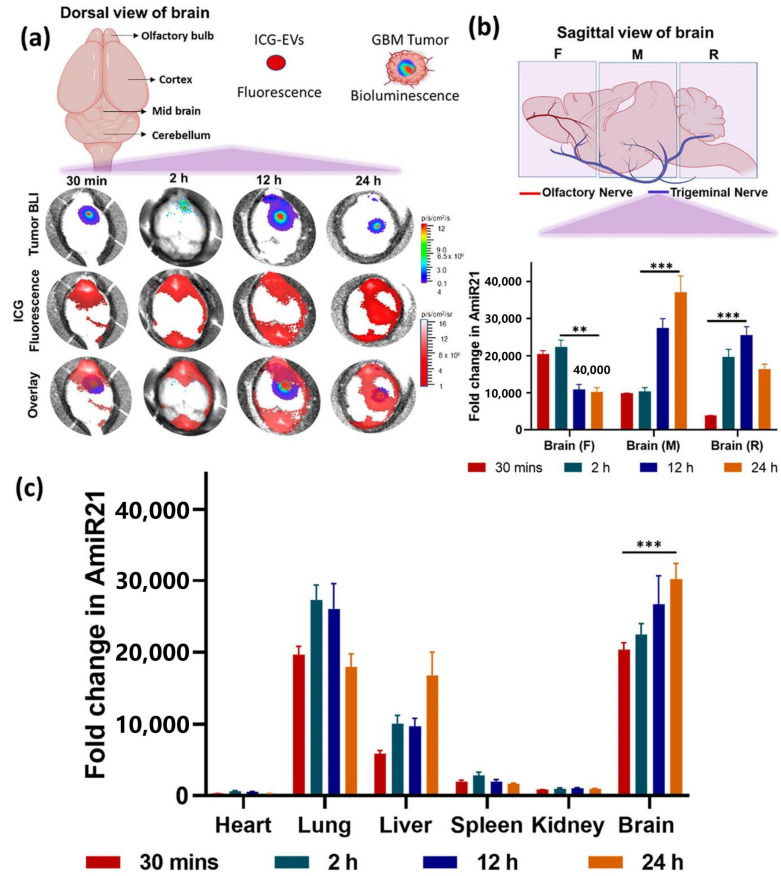
Evaluation of the tumor targeting effect of the mpEVs following intranasal administration. (**a**) Ex vivo imaging of the brain harvested from mice after intranasal administration of ICG-labeled mpEVs at different time points and their intracranial distribution for the tumors. Quantitative estimation of delivered anti-miR-21 in (**b**) forebrain (F), midbrain (M), and rear/hindbrain (R) as well as (**c**) other organs at different time points after a single intranasal dose. ** *p* < 0.01, *** *p* < 0.001. (Reprinted with permission from ref. [61], Copyright 2021, American Chemical Society).

Liu et al. [60] developed a targeted polymeric nanoparticle functionalized with angiopep-2 peptide to co-deliver anti-miR-21 and miR-124 microRNA into the brain to effectively treat GBM. The obtained nanomedicine Ang-NM@Cy5-miRNC displayed significantly longer blood circulation time (t1/2, β = 52.7 min) compared to that of the free miRNA counterpart (t1/2, β = 5.6 min) and could reach a maximum tumor accumulation (8.6% ID g^−1^%) 4 h post i.v. injection. Animal experiment showed that the co-delivered anti-miR-21 and miR-124 simultaneously regulated the mutant RAS/PI3K/PTEN/AKT signaling pathway in GBM cells, and a robust therapeutic effect was obtained. Wang et al. [61] reported the co-delivery of anti-miRNA-21 and miRNA-100 by CXCR4-engineered microvesicles isolated from neural stem cells for GBM therapy. CXCR4 is short for C-X-C chemokine receptor type 4, and it is an alpha-chemokine receptor specific for stromal-derived-factor-1 (SDF-1), a molecule that is overexpressed in GBM cells. The obtained microRNA-loaded exosomes (mpEVs) were intranasally administered into the mouse tumor model, and they could traffic across the nasal epithelia, bypass the BBB into the intracranial compartment, and consequently target GBM for miRNA delivery. The delivered miRNAs sensitized GBM cells to TMZ, resulting in prominent tumor regression, and improved the overall survival of tumor-bearing mice (Figure 4).

### 2.4. Other RNA-Based Nano-Therapeutics for GBM

Short hairpin RNAs (shRNAs) are small, synthetic, double-stranded RNA molecules connected by a hairpin loop that can be used to knock down target genes via RNAi. shRNAs are processed similarly to precursor microRNAs through the endogenous RNAi pathway of transfected cells [62]. The shRNA-based knockdown approach has recently been used to induce gene knockdowns in mammalian cells for disease therapy. CD163 is a 130-kDa membrane protein belonging to the scavenger receptor cysteine-rich (SRCR) family class B domains. It has been reported that CD163 expression is elevated in GBM cells, especially glioma stem cells, and inversely correlated with survival times [63]. Liu et al. [64] synthesized a pH-sensitive DSPE-cRGD-Hz-PEG2000 to form an environmentally self-adaptative nanoliposome for brain-targeted co-delivery of shCD163 and TMZ to treat GBM. The obtained nanoparticles (shCD163/DOX@cRGD-DDD Lip) possessed long blood circulation and efficiently crossed the BBB, benefitting from the PEG shell in the physiological pH conditions. When reached in the tumor site, the weakly acidic pH triggers the detachment of the PEG shell from the nanoparticles and the exposure of the tumor-targeting ligand cRGD, which enhances tumor uptake. shCD163/DOX@cRGD-DDD Lip was able to impair the cell cycle and stemness of GBM cells thanks to the toxicity of DOX, inhibiting the activity of the CD163 pathway. With systemic administration, the nanoparticles efficiently disturbed the progression of the tumors and prevented the recurrence of gliomas after resection.

**Figure 5:**
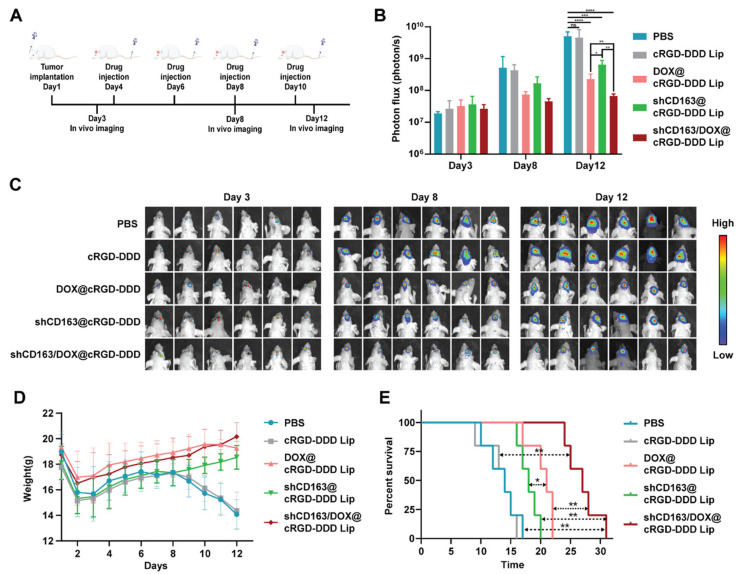
Evaluation of the anti-tumor effect of shCD163/DOX@cRGD-DDD Lips in GL 261 orthotopic GBM tumor models. (**A**) Illustration of the experiment timeline. (**B**) Bioluminescence signal intensity of the tumors in different groups. (**C**) Bioluminescence images of the tumor-bearing mice from each group. Body weight changes and (**D**,**E**) Kaplan−Meier survival curve of mice in each group. * *p* < 0.05, ** *p* < 0.01, *** *p* < 0.001, **** *p* < 0.0001. (Reprinted with permission from ref. [66], Copyright 2021, American Chemical Society).

Antisense oligonucleotides (ASOs) are short (~18–30 nucleotides), synthetic, single-stranded nucleotide sequences that can be employed to modulate gene expression via various mechanisms (e.g., RNase H1-mediated degradation and RNA steric hindrance) [65]. Adamus et al. [66] reported that exosomes secreted from the neural stem cells (NSC) could effectively deliver ASO to target oncogenic STAT3 protein into the glioma microenvironment for tumor therapy. The STAT3 ASO was first conjugated with an immunostimulant CPG to facilitate the entry of the ASO into NSC via the scavenger-receptor-mediated endocytosis mechanism. This strategy does not require stable expression of RNA cargo or ex vivo manipulation, such as electroporation or lipofection, which often fail to efficiently load genetic nucleotide sequences into exosomes. When peritumorally injected into the brain, the obtained exosomes CpG-STAT3-ASO-Cy3 displayed more efficient targeted delivery toward the tumor-associated myeloid cells, such as microglia, macrophages, and MDSCs, compared with the injection of oligonucleotide alone. In an orthotopic GL261 tumor mouse model, efficient antitumor effects of the ASO-loaded exosomes were obtained (Figure 5).

## 3. Conclusions and Future Perspectives

GBM is a very aggressive brain cancer that remains incurable despite the improvements in cancer detection and therapy strategies as well the medical equipment. The conventional surgical resection facilitated by the chemo-/radiotherapy method has limited efficacy for GBM, and the development of resistance and/or tumor recurrence is almost inevitable. Novel therapeutic strategies that can successfully and efficiently block tumor progression are urgently needed. RNA-based GBM gene therapy displays numerous advantages over conventional therapy: (1) It is target-specific and can target the un-druggable therapeutic targets for therapy; (2) it possesses the potential to tackle the challenges, e.g., chemo-/radiotherapy resistance, recurrence, and incurable stem cell-like tumor cells, that confront the conventional methods; and (3) it has high selectivity toward the target genes that are specific to tumors, which would reduce side effects. Additionally, RNA-based therapeutics can be applied in the other newly emerging GBM therapies such as immunotherapy and targeted molecular therapy (e.g., gene-editing therapy). For example, the blooming of RNA-based cancer vaccines has significantly promoted the development of GBM immunotherapy, as they offer the flexibility of targeting multiple tumor-specific epitopes without the risk of genomic integration (DNA vaccines) and without the complexity of human leukocyte antigen-restricted epitopes (peptide vaccines) as well as a short development cycle and cost effectiveness. Gene-editing systems such as CRISPR/Cas have shown great potential in cancer therapy. In this system, Cas enzymes preloaded with sgRNAs targeted at the cancer-related DNA site are delivered into the tumor cells to precisely manipulate the gene editing. From a future development view, using the corresponding RNA techniques (e.g., mRNA) instead of Cas proteins may pave the development and research process of CRISPR/Cas gene-editing systems. Moreover, engineered cell-based therapies are also being explored as promising glioblastoma therapeutics. RNA-based techniques have been widely investigated in GBM cell therapy, e.g., construction of GBM DC vaccines, CAR-T cells, and functional neuronal stem cells.

An important consideration in the application of gene therapy for GBM is the design of appropriate transfection agents and carriers for tumor-targeted delivery of the exogenous RNA molecules. Artificially engineered nanomedicines with the abundance and multifunction of nanomaterials, ease of functionalization with targeting ligands, high RNA loading and transfection efficiency, and low immunotoxicity and insertional mutagenesis risk compared to the viral vectors have attracted enormous research interest in the area of GBM gene therapy. In this review, we have outlined the recent advances in the engineering of non-viral nanomedicines for brain-targeted delivery of RNA drugs for GBM therapy, with an emphasis on the BBB-penetration strategies and the therapeutic mechanisms of these nanodrugs for tackling the challenges confronting the current GBM therapy methods.

Despite the promising prospects in achieving highly efficient GBM gene therapy, few RNA–nanoparticle systems have been tested in clinical trials for GBM therapy. Data from the ClinicalTrial. gov. reveals that only one RNA interference-based nanotherapeutics NU-0129 was tested in an early phase 1 clinical trial (NCT03020017). The obstacles preventing the clinical translation of RNA-based GBM nanotherapeutics involve the difficulty of efficiently tumor-targeted delivery of such gene drugs, off-target binding, and sequence-induced toxicity. The rational design of advanced nanocarriers provides excellent BBB transport for GBM targeting, but the nonspecific biodistribution of the nanoparticles cannot be avoided, and most of the developed nanodrugs for GBM therapy lack systemic study of their pharmacokinetics and biodistribution in clinical trials, which induces potential risks of the alteration of the somatic cells gene expression of the patients who received the nanomedicine treatment. Moreover, several other challenges also need to be considered before the potential of the engineered RNA-based nanotherapeutics is fully realized. First, safety concerns regarding nonspecific tissue accumulation of the nanoparticulate gene delivery system and its long-term toxicity in the organism should be fully explored. Second is the economic large-scale production of the RNA drug-loaded nanomedicine. On the one hand, the synthesis and modification of RNA oligonucleotides with optimal activities is cumbersome and expensive. On the other hand, RNA molecules, especially mRNAs, have poor stability and easily degrade in solution. Thus, it is necessary to develop good manufacturing-practice-compliant equipment, facilities, and procedures for the cost-effective production of these RNA-loaded nanomedicines. Third, deep understanding of the biological pathways of these nanomedicines is needed for achieving more potent tumor targeting and therapeutic effects. Fourth, the ethical issues of RNA gene therapy should be considered. For example, will the high costs of gene therapy make it available only to the wealthy? Will the gene expression alteration in somatic cells result in unwanted heritable side effects? At present, there is no clear evidence that the benefits of gene therapy outweigh the side effects to human beings.

In summary, RNA-based nanoparticles are highly promising for GBM therapy not only as a therapeutic drug to inhibit tumor progression but also as a strategy to integrate with newly emerging and promising therapies such as GBM vaccines, CAR-T cells, as well as functional neuronal stem cells to achieve efficient GBM therapy. Despite the challenges and disappointing clinical results that exist in developing RNA-based nanotherapeutics for GBM, the pursuit of this approach remains warranted due to the significant therapeutic potential and rapid pace of progress in the field.

## Data Availability

Not applicable.
